# Re-building relationships after a spinal cord injury: experiences of family caregivers and care recipients

**DOI:** 10.1186/s12883-019-1347-x

**Published:** 2019-06-08

**Authors:** Gaya Jeyathevan, Jill I. Cameron, B. Catharine Craven, Sarah E. P. Munce, Susan B. Jaglal

**Affiliations:** 1 Institute of Health Policy, Management and Evaluation, University of Toronto, Toronto, Ontario Canada; 2 Neural Engineering and Therapeutics Team, KITE, Toronto Rehab – University Health Network, Toronto, Ontario Canada; 3 Department of Occupational Science and Occupational Therapy, Toronto, Ontario Canada; 4 Department of Physical Therapy, University of Toronto, Toronto, Ontario Canada; 5 Department of Medicine, Division of Physical Medicine and Rehabilitation, University of Toronto, Toronto, Ontario Canada

**Keywords:** Family caregiving, Spinal cord injury, Qualitative, Relationships, Dyads

## Abstract

**Background:**

Following spinal cord injury (SCI), family members are often called upon to undertake the caregiving role. This change in the nature of the relationship between the individuals with SCI and their families can lead to emotional, psychological, and relationship challenges. There is limited research on how individuals with SCI and their family caregivers adapt to their new lives post-injury, or on which dyadic coping strategies are used to maintain relationships. Thus, the objectives of this study were to obtain an in-depth understanding of 1) the experiences and challenges within a caregiving relationship post-SCI among spouses, as well as parents and adult children; and 2) the coping strategies used by caregivers and care recipients to maintain/rebuild their relationships.

**Methods:**

A qualitative descriptive approach with an exploratory design was used. Semi-structured face-to-face and telephone interviews were conducted. Thematic analysis was used to identify key themes arising from individuals with SCI’s (*n* = 19) and their family caregivers’ (*n* = 15) experiences.

**Results:**

Individuals with SCI and family caregivers spoke in-depth and openly about their experiences and challenges post-injury, with two emerging themes (including subsequent sub-themes). The first theme of deterioration of relationship, which reflects the challenges experienced/factors that contributed to disintegration in a relationship post-injury, included: protective behaviours, asymmetrical dependency, loss of sex and intimacy, and difficulty adapting. The second theme of re-building/maintaining the relationship, which reflects the strategies used by dyads to adjust to the changes within the relationship brought upon by the injury, included: interdependence, shifting commonalities, adding creativity into routine, and creating a new normal.

**Conclusions:**

These findings should alert healthcare professionals and peer support groups as to the need for possible education and training (e.g., coping strategies, communication skills training) as well as counseling prior to discharge to assist individuals with SCI and family caregivers with adaptation to a new life post-injury.

## Background

Following spinal cord injury (SCI), spouses and other family members are often called upon to undertake the caregiving role [1]. Changes in the nature of the relationship (e.g., role change from spouse to caregiver) between the individuals with SCI and their families can challenge the family physically, psychologically, and socially [2, 3]. Comparable to the individuals with SCI, the family caregivers are often required to reconstruct their lives, as well start an "unexpected career" to provide support with activities of daily living (ADLs), personal care, and emotional support to their family member with a disability [4, 5].

The stress associated with assuming the caregiving role [6] can influence the adjustment of individuals with SCI and their families [7]. Cleveland [8] determined that tasks associated with caring for an individual with SCI were often unevenly distributed amongst family members, thereby resulting in increased stress in communication, family unity, family power structure, and interpersonal relationships. In a recent descriptive cross-sectional study assessing the relationship between sociodemographic factors and level of burden experienced among caregivers of individuals with SCI, it was reported that parents experienced significantly more burden undertaking the caregiver role in comparison to spousal caregivers [9]. Indeed, parents who are caregivers (i.e., of adult individuals with SCI) are usually older than spousal caregivers, and therefore, experience greater physical burden [9] due to their own functional limitations [10]. With respect to partner relationships, DeVivo and Fine [11] explored the impact of SCI on the marital status of 276 injured individuals for a 3-year period post-injury. They found a significantly higher incidence of divorce and separation during the first 3 years after the injury, indicating it was very demanding trying to rebuild daily life and relationships during the early stage of the injury [11]. The authors also reported that women with SCI are more likely to be divorced than men. In a later study, DeVivo and Richards [12] determined that among the individuals who were married at the time of their injury, 81% were still married 5 years later. In another study conducted by Kreuter and colleagues [13], findings showed that challenges in adapting to new physical functions, unwillingness to live with the injured individual, and difficulties in maintaining the relationship resulted in divorce post-injury. The authors highlighted the importance of the partners’ mutual support and the significance of maintaining a marriage/relationship [13].

Emerging literature on dyadic coping between caregivers and care recipients provides insight into how couples manage health, relational, and psychosocial issues [14–16]. Among those who had traumatic brain injury (TBI), Adams and Dahdah [17] reported that problem-focused, emotion-focused and avoidant coping were some strategies used by TBI survivors and their primary caregivers to cope with home life and ADLs. However, it is imperative to recognize that, within each relationship, couples may experience the effects of the illness or injury differently [18, 19]. Another recent study by Robinson-Smith and colleagues [20] took a dyadic approach to understanding the impact of stroke on relationships to identify dyadic coping strategies that may provide guidance for a focused nursing intervention to support post-stroke couples. The authors reported that dyadic coping strategies such as focusing on the present, positive reframing and focusing on reasonable goals should be incorporated in cognitive and communication coping interventions to enhance coping skills and overall well-being of couples post-stroke. Although these studies provide valuable insight into the nature of caregiving relationships, the extent to which these findings can be generalized to individuals with SCI and their family caregivers is unknown. Indeed, providing care to individuals with SCI is distinct from other illnesses and injuries due to the unique challenges of providing care related to the complications associated with SCI (e.g., bladder and bowel problems, and pressure injury management [21]) and the longer duration of their caregiving period [22].

While previous studies have focused on the impact of SCI on relationships [7, 23, 24], these studies have used quantitative methods to examine marital status, long-term marital survival, and the impact of SCI on the relationship from the perspective of the individual with SCI [5]. Furthermore, these studies highlighted the negative impact of SCI on relationships including, difficulty reestablishing life as a couple, being socially active as a couple, and communicating feelings [7, 23, 24]. There is limited research on how individuals with SCI and their family caregivers adapt to their new lives post-injury, or on which dyadic coping strategies are used to maintain relationships. Furthermore, when an individual is younger (i.e., young adult) and not married, the role of caregiving most often falls to the parents. Yet, there is a paucity of published literature on parents as caregivers and the impact of SCI on the parent-child relationship. As a result, there is an increased need for qualitative research examining partner/parent relationships post-injury. Thus, the objectives of this paper were to obtain an in-depth understanding of 1) the experiences and challenges within a caregiving relationship post-SCI among spouses, as well as parents and adult children; and 2) the coping strategies used by caregivers and care recipients to maintain/rebuild their relationships.

## Methods

### Design

A qualitative descriptive study with an exploratory design was undertaken [25, 26] as there are limited studies examining the relationship between individuals with SCI and their family caregivers. This is the most appropriate qualitative approach to gather in depth information about the lived experiences of the caregivers and care recipients within their “natural context” [27]. Furthermore, this allowed us to address gaps in our overall understanding of challenges in relationships post-injury, provide a new perspective on the variation between parent-child and spousal interactions and coping strategies, and gain insights about a phenomenon that has rarely been studied.

The data used in the current study were based on qualitative interviews conducted by the primary author (GJ) for a larger study exploring the support needs of family caregivers of individuals with SCI. During the interviews, topics related to the impact of SCI on relationships emerged in the dialogue of the participants. The insights that emerged during the early interviews (first 3 interviews) were then introduced to the 31 subsequent interviews and new themes were identified based on the participants’ responses. For example, when asked about the facilitators and barriers to caregiving (consistent with the original interview guide), emerging issues such as relationship challenges experienced and coping strategies devised as dyads post-discharge were frequently mentioned by both the individuals with SCI and family caregivers. Research ethics approval was obtained from the University Health Network (16–5093.2) and the University of Toronto (Protocol Reference #33202). All participants provided informed verbal consent at the time of the interview.

### Participants and recruitment

Considering the potential synergistic effects of caregiving whereby caregivers and care recipients mutually affect each other [22], the current study included both the individuals with SCI and their family caregivers. Individuals with SCI who are living in the community across Canada and their family caregivers were recruited from a list of participants (who had previously agreed to be contacted for research purposes) from the Rick Hansen Institute SCI Community Survey (RHISCICS); Toronto Rehabilitation Institute (TRI) Lyndhurst Centre- a large outpatient SCI clinic; and Spinal Cord Injury Ontario (SCIO)- a community-based service provider to individuals with SCI. Participants were recruited through: i) a letter of invitation sent via e-mail to participants from the RHISCICS; ii) referral by healthcare professionals at TRI Lyndhurst Centre; and, iii) an online advertisement posted on the SCIO website. Purposive sampling by time since discharge (i.e., within 2 years post-discharge and more than 10 years post-discharge from inpatient rehabilitation) was used in the selection and recruitment of participants [28]. Within the first 2 years post-discharge, there is an initial learning curve for family members in figuring out how to provide support to the individual with SCI. Also during this period, both explicit and implicit needs arise from both the individuals with SCI and family caregivers [29], as well there is a higher incidence of separations and divorces [11]. Conversely, the comorbidities related to aging and the cumulative effects of secondary health conditions associated with SCI, are more apparent at 10 years post-discharge [30], which results in evolving supportive care needs for individuals with SCI and family caregivers over time. Inclusion criteria for individuals with SCI included: 1) were at least 18 years of age; 2) had a spinal cord injury of either traumatic (e.g., fall, motor vehicle accident, sporting accident) or non-traumatic (e.g., cancer, disc degeneration of spine, inflammation, arthritis) etiology; 3) were 3–24 months post-discharge from inpatient rehabilitation or over 10 years post-discharge; and 4) were fluent in English. Family caregivers were recruited through the individuals with SCI and were identified as his/her primary caregiver. Inclusion criteria for family caregivers included: 1) were a spouse/partner or parent of an individual with SCI; 2) described themselves as providing physical and/or psychological support to the individual with SCI; 3) had regular contact with the individual with SCI (i.e., at least weekly face-to-face contact); and 4) were fluent in English. Participant recruitment occurred between August 2016 to April 2017. Recruitment concluded when the study reached data saturation, whereby the information from new interviews became repetitive and no new themes emerged [31].

### Data collection

Data collection included separate, semi-structured interviews through telephone or face-to-face interaction with caregivers and care recipients (please see Table 1 for a list of examples of open-ended questions). Interviews were conducted separately to mitigate any potential power imbalances between the caregivers and care recipients which could affect the experiences they would be willing to share. The interview guides were developed using grey and published literature, and in accordance with a standardized Theoretical Domains Framework (TDF) interview guide [32]. The TDF is an integrative framework consisting of 14 domains and 84 constructs that can be used to guide the design of appropriately targeted interventions or evidence-based programs. The interview guides for both the individuals with SCI and family caregivers were pilot tested with one of the authors experienced in qualitative research methods (JIC) as well as an individual with SCI and his caregiver. Probes or recursive questioning were used during interviews to explore topics in greater depth [33] and to enable the participants to share any experiences they felt were crucial to the study. All telephone and face-to-face interviews were audiotaped, transcribed verbatim, and reviewed for accuracy.

**Table 1:** Examples of Open-Ended Questions from the Interview Guides for Impact of SCI on Relationships

1. Caregiver: Do you feel that your relationship with your partner/child changed after his/her injury?	
2. Caregiver: How did you and your family member handle sex and intimacy during the early stage of injury and have there been any changes now (please explain)?	
3. Caregiver: How does providing care to your family member impact your other roles as a mother/father, spouse, active community member, etc.?	
4. Care recipient: Can you please tell me how you and your family member adjusted to the changes after discharge (i.e., immediately after discharge and/or past the 10 years)?	
5. Care recipient: From your perspective, what do you think are some benefits of your family member being the primary caregiver?	
6. Care recipient: How do you think providing care to you affects your family member emotionally (positively and negatively)?	
a. Probing: How do you help your family member cope with these negative emotions when providing care to you?	

### Data analysis

The inductive thematic analysis procedures of open and axial coding, and comprehensive memo writing constituted the basic analytic techniques [34]. Qualitative software, NVivo 10 [35], was used to organize and analyze the data. Considering the potential variability in the level of support provided when taking care of an individual with SCI by different family members, analysis was stratified based on caregiver relationship (i.e., spouses and parents). Starting with open coding, a subset of the transcripts was initially coded by the primary author (GJ), and a ‘description’ was assigned for each event, idea, or phenomena discussed by each participant using an inductive approach. For example, excerpts of the transcripts that described the challenges experienced that caused a deterioration in relationships post-injury, as well as strategies (i.e., behaviours and actions) used by caregivers and care recipients in maintaining/re-building their relationships were initially examined. Overlapping and contrasting data between care recipients and caregivers were then grouped. This ensured a global perspective that is more than the sum of the individual accounts. Subsequently, codes were clustered into categories and apparent themes were identified. Two independent researchers further coded the same transcripts to enhance the reflexivity and rigor of the study. In addition, four members of the research team had ongoing peer debriefing meetings to discuss the analysis and interpretation of data to enhance trustworthiness and credibility.

## Results

Thirty-four interviews were conducted, including 19 individuals with SCI and 15 family caregivers (9 spouses/partners, 6 parents). Among the 34 participants, 26 individuals were in dyads (13 caregiver-care recipient dyads in total), and 8 individuals participated on their own (2 caregivers, 6 care recipients). All participants discussed changes to their relationships and were included in the analyses. Characteristics of the individuals with SCI and family caregivers are reported in Table 2. While most (75%) of the family caregivers had been providing support for more than 10 years, the remaining (25%) had assumed the caregiving role for only 6 months to 2 years. Care recipients and caregivers had regular contact (26 lived together, and 8 saw each other at least weekly). One care recipient had frequent contact with her caregiver (i.e., lived together) until their recent divorce. Interviews lasted between 45 min to 2 h. To secure anonymity, quotations representing the various themes include only the participants’ group (i.e., care recipient or caregiver).

**Table 2:** Characteristics of Participants in the Study

Characteristics of Individuals with SCI	*N* = 19
Sex	
Male	13 (68%)
Female	6 (32%)
Time since discharge from inpatient rehabilitation (years)	
< 2 years post-discharge	4 (21%)
> 10 years post-discharge	15 79%)
Level of injury	
Paraplegia	11 (58%)
Tetraplegia	8 (42%)
Relationship to family caregiver	
Spouse/Partner	11 (58%)
Child	8 (42%)
Age (range)	22–65
Characteristics of Family Caregivers	*N* = 15
Sex	
Male	3 (20%)
Female	12 (80%)
Employment status	
Employed	8 (53%)
Unemployed/retired	7 (47%)
Relationship to individual with SCI	
Spouse/Partner	9 (60%)
Parent	6 (40%)
Age (range)	41–82

**Fig. 1:**
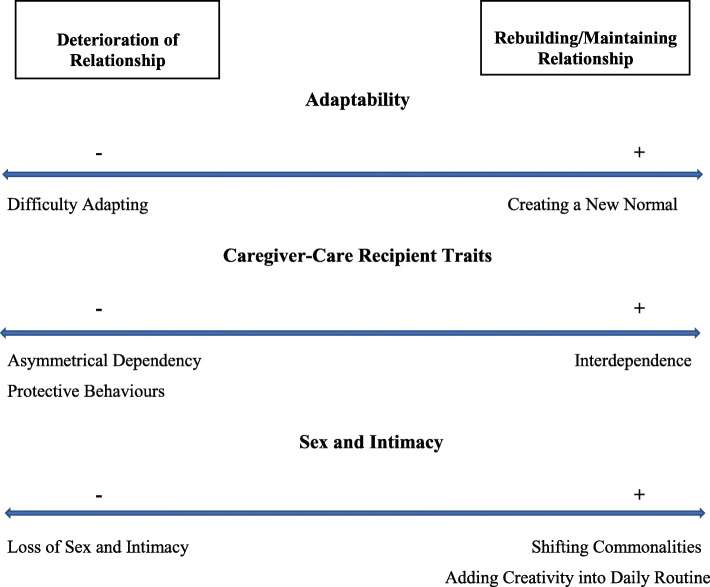
Caregiver-Care Recipient Interaction/Behaviour Spectrum: Identified Factors of Deterioration of Relationship and Associated Strategies in Rebuilding/Maintaining Relationship

Individuals with SCI and family caregivers spoke in-depth and openly about their experiences and challenges post-injury, with two emerging themes (including subsequent sub-themes): 1) deterioration of relationship- this reflects the challenges experienced/factors that contributed to disintegration in a relationship post-injury; and 2) re-building/maintaining the relationship- this reflects the strategies used by dyads to cope with the changes within the relationship brought upon by the injury. Figure 1 portrays a dyadic coping spectrum composed of the identified challenges experienced/factors that contributed to disintegration in a relationship post-injury and the corresponding strategies that caregivers and care recipients used in coping with these challenges to maintain/rebuild their relationships post-injury.

### Deterioration of relationship

#### Difficulty adapting

Both the individuals with SCI and their family caregivers reported difficulty adapting to the injury post-discharge, and this caused several negative effects, emotionally and physically, within the relationships. Some participants stated that rather than positively coping and creating a new life with the injury as a dyadic unit, they were focused on a rapid recovery. One recently divorced care recipient explained how not being able to adapt to the new life post-injury, coupled with a break-down in communication with her husband led to the dissolution of her marriage:

*“I think we really didn’t adjust to it, we just kind of went around it. We just kind of tried to do everything we did before. I got injured, and tried to avoid the subject as much as possible, it felt like. We never … there was always that big elephant in the room, but nobody really wanted to talk about it because nobody wanted … I didn’t want my feelings to get hurt, and I didn’t want his feelings to get hurt. It was always, you almost never wanted to accept that you were going to be in … even ten years later, you’re still like, well, no … 15 years later, no, no, I’m still going to walk, that magic pill is going to be available. It was just never accepted that I would be in the chair for the rest of my life, so it was almost like, never addressed.”* (care recipient 7, wife)

Some participants went on to add that due to not being able to emotionally cope with living with the injury, they faced difficulty in physically adapting to the injury as well (i.e., inability to emotionally cope to injury hindered willingness to be physically and socially active as a dyad).

#### Protective behaviours

When family members were involved in the caregiving process, they often engaged in protective behaviours, whereby they safeguarded the other individual’s emotional or physical wellbeing. This sometimes resulted in detrimental effects on the individual (i.e., care recipient or caregiver) and within the relationship. Several participants, particularly within spousal relationships, described their experiences of using protective buffering within their caregiving relationships. Participants reported hiding worries and concerns and yielding to the other person in an effort to reduce the other person’s upset and burden. For example, to protect her husband (caregiver) and their marriage, one care recipient mentioned how she set aside her own hardships (physical and emotional challenges) and attempted to make her physical pain less discernible and less burdensome on her caregiver:

*“I made it look like, you know what, I’m totally okay, don’t worry about me, everything is fine. I almost never wanted to complain about anything to him [caregiver], thinking, oh my god, this is too much already, and if I even tell him that, oh my god, my hands hurt today or something, and I can’t do something, I’m just putting more pressure on a person. I usually would never…I would just kind of deal with the pain, and move on because I didn’t want to put any more burden on that person…I think he got frustrated with me a lot, not telling him what was wrong or something. I think there was a lot of frustration on his part, on knowing what to do or how to help because I wasn’t co-operating, and letting him know what kind of help I needed.”* (care recipient 1, wife)

Similarly, caregivers mentioned they would occasionally hide their distress related to caregiving from their family members who are injured to protect their feelings of guilt for requiring daily assistance.

Care recipients also reported that caregivers, particularly parent caregivers, were sometimes over-protective. Several added that caregivers usually developed a vigilant attitude or some even underestimated the care recipients’ abilities, thus assuming responsibility for many daily tasks. Some care recipients went on to mention that this protective behaviour diminished their feelings of self-efficacy and autonomy, and this, in turn, often led to resentment within the relationship.

#### Asymmetrical dependency

Although the majority of individuals with SCI reported they required at least some assistance from their family caregivers, there were several participants who relied heavily on their caregivers to meet their physical needs. Some participants mentioned that this asymmetrical dependency within their relationships had resulted in either or both care recipient and caregiver losing their identities and freedom. Some care recipients had a general expectation of their caregivers to meet all their daily needs, and this resulted in caregivers, who are living under the burden of these expectations, to exhibit signs of frustration and withdrawal. One distressed caregiver explained how asymmetrical dependency portrayed by her injured husband affects her psychological wellbeing:

*“He [care recipient] wants to occupy [exploit] me. He wants to occupy me, and what he can do himself, he wants me to do it, yeah. But after, in 2006, I [became] exhausted. I told him that I will not be with him, and then I went back [to parents’] home.”* (caregiver 10, wife)

On the other hand, some caregivers showed dysfunctional helping behaviours and provided constant round-the-clock care to the injured individuals. Caregivers’ protective and caring behaviours developed into more dependency-inducing behaviours (i.e., where caregiver does for the care recipient what he/she should be doing for him/herself), and this contributed to a co-dependent relationship. Examples of dependency-inducing behaviours include completely undertaking basic self-care and instrumental tasks for injured individuals in situations where they are capable of acting independently (e.g., dressing, preparing meals, cleaning, etc.). This behaviour was more prominent in parent-child dyads, particularly as the participants reported it stemmed from a sense of obligation or duty to provide care as a parent:

*“When you’re in a wheelchair, people will do everything for you, if you let them. Some people who are injured are more than happy to take advantage of that. Especially parents. I’ve noticed this over the years. It’s really hard for a parent to see their child in that situation. Anybody who sees their loved one struggling, they have a really hard time with that and they want to help them out as much as possible, which means they want to do things for them...We’re so focused on the injured individual that we don’t realize what the caregivers are going through. Again, they’re questioning, am I pushing too hard? Should I back off? He’s frustrated, should I take over or should I let him work through that? All of that creates all kinds of anxiety and that anxiety day over day over day really starts to wear on a person. By the time the person, the caregiver, realizes it, they’re relationship with that individual is probably affected in a negative way.”* (care recipient 14, son)

Several participants noted that not only was this type of behaviour creating an asymmetrical dependency within the relationship, but also reduced the care recipient’s self-efficacy and autonomy.

#### Loss of sex and intimacy

Several participants in romantic relationships mentioned they experienced loss of sex and intimacy post-injury. Role change from spouse to caregivers was reported by dyads as a key factor in changing the dynamics of the relationship and caused a strain in the couples’ relationships. Several participants added that due to the psychological distress associated with providing care, particularly, the constant care provision for individuals with tetraplegia, they could not connect intimately with the care recipients. As one care recipient put it: *“As much as she gives me care she is more upset, so it’s more difficult and bonding is more loose* (care recipient 10, husband).” Some individuals described initial apprehension and concerns about initiating sexual intimacy with their partners. Other participants reported that a loss of spontaneity within their relationships interfered with their ability to bond and build intimacy. Individuals felt the constant routine associated with providing care left no room for creativity in their romantic relationships. One care recipient explained how lack of spontaneity resulted in her and her caregiving spouse’s recent divorce:

*“I think you just get into a cycle, very repetitive, you just kind of get used to the way things are done, and then you just repeat them every single day, so it’s just kind of forgotten. I think that’s a lot what broke us apart, it was just, he started doing his own stuff, that I wasn’t able to do, and I was left alone a lot. I thought, well, why bother staying…Again, there was no being spontaneous if you felt in the mood or whatever, it was very calculated, okay, well, it’s going to happen. I guess I could not be spontaneous, so everything was mapped out. That it’s almost like, I don’t know even if it’s worth it anymore. It’s just like, same thing over and over, there is no variations to it, I’m going to say.”* (care recipient 7, wife)

### Re-building/maintaining the relationship

#### Creating a new normal

Creating a “new normal” was described as progressively establishing a new routine as a dyad with a focus on the individual with SCI’s abilities. Both care recipients and caregivers noted that life does not return to their former routines post-injury. Therefore, they had to create a new normal, such as developing a new daily routine whereby the injured individuals contributed to household responsibilities according to their level of function. For example, several care recipients who were originally the primary earners prior to the injury had returned to work, but as part-time and/or with new jobs accommodating their capabilities so as to reduce the financial strain on the caregivers. As one parent caregiver proudly stated:

*“…he says, mom, I am not going to be on this the rest of my life. I can work. Yes, even if I’m in a wheelchair the rest of my life, I am not going to be on government assistance for the rest of my life. If I have to do a desk job, which was not his ideal job…like I say, he wanted to be a police officer and be out doing things. But if that doesn’t work out, he said I’m going to have a job.”* (caregiver 19, mother)

Several participants also acknowledged that undertaking household chores (e.g., cooking, laundry, etc.) and other daily activities (e.g., providing care to young children) that were not their former roles allowed them to create a new normal as a family. One caregiver described how although his injured wife is not physically able to participate in leisure activities as before, they were able to reach a new normal as well as redefine their relationship by discovering alternative ways to be socially active as a couple:

*“…we carried on accordingly. I mean we had to, of course, learn a lot of new aspects in terms of making life acceptable and comfortable as much as possible. So, I think we did kind of a dip in the graph, but we pulled ourselves up again to a level of normality. Of course, it would have been so much easier for [care recipient] to be in good health and we would have done what many of our friends do. But we do it still, in brackets. They travel all over the world. So, do we, except we always have to put the caveat in, well, is it adapted, what are the possibilities of doing a, b, and c. Which when people, for example, go on a cruise, they can book an excursion to Machu Picchu or something, we can’t. Things like that. Those are little things which curtail our mobility, but nevertheless we’re doing it anyway and we look forward to that. I think that allows both of us to not miss out shall we say. It would have to be recalibrated and adapted, but we’re doing it anyway. And that I think is something that is very bonding and very uplifting for both of us.”* (caregiver 6, husband)

Both participant groups further reported that a dual effort was necessary as a dyad in working together towards realistic recovery expectations, utilizing effective communication skills.

#### Interdependence

Although the individuals with SCI felt they were more dependent on their caregivers for assistance with daily activities, both participant groups emphasized the need to follow an interdependent approach to managing their relationships. Participants described interdependence as both individuals within the dyad being mutually reliant on each other (i.e., having equality and balance in how each individual’s needs are met), while being able to maintain their autonomous identities. Both participant groups noted that they needed to address their needs and concerns to understand their roles within the caregiving relationship. For example, most individuals mentioned the need for open communication in caregiving situations whereby the caregiver needed to address caregiving boundaries (i.e., caregiving tasks he/she is willing to and capable of doing) and the care recipient needed to direct care (i.e., how and to what level he/she wants assistance with ADLs). As one care recipient had suggested:

*“You have to allow your caregivers to have an out. Some days they just don’t feel like doing it and other times they don’t want to do what you need done. Some caregivers would be okay with helping you with catheterization and wound care, but bowel care, no thank you, they don’t want anything to do with that. Whereas, another person would be the exact opposite. They have to be honest and up front about where their limits are. That’s something that should be established right at the very beginning…You’ve got to remember, the caregiver is probably somebody very close to the person who’s injured, so they’re going through all that emotion and that sense of loss, how is this going to affect their lives, all that. They’re going through all of that as well. The emotional burden on your caregiver when you’re first injured can be psychological, can be physical, can be a lot of different ways, and that leads to caregiver burnout, that leads to deteriorated relationships and all kinds of other problems. So, right in the beginning, have that discussion. Communication with your caregiver is so important. If they don’t have the communication skills, if their relationship is lacking in any way, this is going to show it. Add the burden of the injury and the caregiving and it’s really hard for a lot of couples to manage that situation.”* (care recipient 13, husband)

Although the issue of directing care and setting boundaries was brought up in parent-child relationships as well, a determined commitment to remain in marriages by having such open communication was mostly expressed by spouses. Where physical assistance was required, several individuals were able to share household tasks, dividing them based on what the individual with SCI wanted and was able to do: *“I do a little bit of laundry, like I put the stuff in the washer and transfer it to the dryer, but she folds the clothes because I really hate folding clothes. If she has a faucet leak, then I’ll fix that, so there’s stuff like that. So, we trade back and forth that way.”* (care recipient 9, husband).

It was also noted by both care recipients and caregivers, particularly spouses/partners, that just as it is important to be mutually supportive in a caregiving situation, it is also necessary to maintain autonomy. Participants explained that by maintaining autonomy, they were able to pursue their own interests and passions. One caregiver described the successful interdependent interaction between himself and his injured wife whereby they followed their own interests while also coming together to participate in dyadic activities:

*“…or down in the country, she can just do that by herself and see her friends and meet deadlines, and I can go and play tennis at the club etcetera. Although we do in the summer, we spend about three or four days in the country and less in the city…She found a place which has adapted safari vehicle, so away we go…We’re together but not necessarily that overpowering in any way, not too dependent on the other. It’s just a matter of we’re here for each other. This is perhaps blowing one’s own horn, but we’ve hardly, if ever, had arguments. That’s almost the earmark of our marriage.”* (caregiver 6, husband)

Some caregivers further added that in order to ensure an interdependent relationship, they had to learn when to step back from caregiving tasks, as well as have open communication with their injured family member. Furthermore, numerous participants, both caregivers and care recipients, emphasized the need for more relationship-based education, including communication techniques and skills that are necessary to establish an interdependent caregiving relationship.

#### Shifting commonalities

Both care recipients and caregivers had noted that due to the changes in psychosocial functioning post-injury, considerable adaptation was necessary as a dyad to re-build their relationships. Several participants reported that they shifted commonalities whereby the dyads changed focus of pre-injury common interests to adapt to the new life post-injury (i.e., discontinued pre-injury activities and focused on new activities to do as a dyad post-injury). The majority of individuals enhanced intimacy through a change of focus on shared activities, rather than focusing on the loss of sex: *“I think you just have to re-shift it to commonalities of things that you enjoy to do together, such as watching movies, and baseball. Re-shifting to commonalities in other areas”* (caregiver 9, wife). Several caregivers had added that they had shifted commonalities based on the care recipients’ abilities and interests:

*“He loves music, he plays the guitar really well. And that’s sometimes what I do too, when he comes to my place. He has his guitar here, so I’ll get it out and say how about singing me some music? And he’ll say, okay. And sometimes he writes songs and whatnot too, and I sit and I listen to them and the whole bit, so it’s things like that.”* (caregiver13, wife)

Shifting commonalities ensured that individuals with SCI did not feel rejected or isolated, and allowed the dyads, particularly spouses, to increase their opportunities for intimacy.

#### Adding creativity into daily routine

Using creativity in caregiving activities was often reported by caregivers. However, a few participants who were spouses reported that it was necessary to incorporate imaginative strategies in daily caregiving tasks, not only to effectively complete care-related tasks, but also to engage with the care recipient intimately. Adding creativity into daily routine helped spouses to rekindle romance in marriages. One specific strategy used in spousal relationships was incorporating role play in day-to-day activities:

*“One of the things you develop with your partner is you like to role play. One of the common role plays is nurse-patient, for example. That’s how you could incorporate your disability into your sex life. I’m just trying to give an example of how you can incorporate some of the caregiving into a more intimate act…At the same time, she’s caregiving, but it kind of changes to more of a romantic experience. I’m not saying this works for everybody, I’m just saying, keep an open mind, because by doing that you’re reducing the caregiving aspect of it and it’s more of a different way of being intimate…The way I’m talking to you, the ideas that I’m talking about now, about using caregiving in intimacy, that was not talked about in rehab at all. This is stuff that I’ve learned through the years. I think in the beginning, something like that, just giving a couple that idea that your caregiving doesn’t necessarily mean that it’s a medical environment. It might take like a year before you can get into this kind of thinking, like this isn’t probably something you’d do the first time, but just to have it out there that this is a way we can lessen the caregiving role and add to the more intimacy.”* (care recipient 16, husband)

## Discussion

The current study aimed to understand the factors that may challenge the stability of relationships post-injury, and coping strategies used by care recipients and caregivers in maintaining/re-building their relationships. The factors that challenged relationship stability include: protective behaviours, asymmetrical dependency, loss of sex and intimacy, and difficulty adapting. The coping strategies used by care recipients and caregivers to maintain/re-build their relationships include: interdependence, shifting commonalities, adding creativity into routine, and creating a new normal. To the best of our knowledge, this is one of the few studies to provide insight into the impact of SCI on parent-child caregiving relationships. In addition, the majority of studies have focused on the negative impact of SCI on relationships [6, 7, 13]; this is the first study within the SCI population to identify various dyadic coping strategies used by care recipients and caregivers to maintain/re-build their relationships. Indeed, the manner in which caregivers and care recipients interact and cope post-injury can be visualized across a spectrum. Figure 1 presents a dyadic coping spectrum consisting of the identified challenges experienced/factors that challenge relationship stability and corresponding strategies that care recipients and caregivers used in coping with these challenges/changes to maintain/rebuild their relationships post-injury. These are discussed below in the context of the existing literature.

Studies have frequently documented that individuals with SCI, particularly those who are tetraplegic, rely on their family members for support with daily activities [36, 37]. As such, the present findings extend those of previous authors in that our participants did acknowledge a presence of asymmetrical dependency within their relationships. Our study corroborated DeSanto-Madeya’s [7] finding that such asymmetrical dependency created a sense of loss for individuals with SCI and their family caregivers. Our findings further indicate that caregivers who were burdened by over-dependency by their injured family member portrayed signs of frustration and withdrawal, which led to emotional detachment and reduced likelihood of intimacy. This was also noted by Milligan and Neufeldt [38] who described that an individual with SCI who aims to minimize the impact of his/her injury on the caregiving partner would make a more “attractive candidate” for a long-term relationship compared to an individual who excessively relies on his/her partner.

In addition, the current study further highlighted the detrimental effects within relationships due to co-dependent behaviours exhibited by caregiver-care recipient dyads. A mutually-fed escalation occurred between dyads whereby caregivers’ protective attitudes (due to underestimating the injured individual’s functional ability or concerns for safety) resulted in dependency-inducing behaviours that may have contributed to care recipients being more dependent. This spiral causes the dyad’s interactions to become rigid, and often led to resentment within the caregiving relationship. Interestingly, this co-dependent behaviour identified in our study can be explained by Blalock’s [39] nonrecursive model of caregiving and dependency. The model explains that dependency-inducing behaviours by informal caregivers are a function of care recipient dependency needs. Indeed, care recipient dependency needs activate the cycle that can lead to more dependency-inducing behaviours by caregivers who begin to “do for” care recipients [39]. This, consequently, may reinforce dependent behaviours by care recipients, and a cycle of care recipient helplessness followed by caregiver strain may be prompted. Our findings further indicate that such dependency-inducing behaviours are more prevalent in parent-child caregiving relationships. Although the participants in our study had stated this behaviour was due to a sense of obligation as a parent to provide assistance to their injured child, Young [40] further attributed feelings of helplessness and guilt as factors of parents usually continuing dependency-inducing behaviours. Moreover, while several individuals expressed profound distress within their relationships, some talked of how they had adjusted to the changes and had followed an interdependent approach to re-building their relationships. Consistent with our findings, another study by Chan [41] also found that sharing household responsibilities based on what the individual with SCI is capable of doing (i.e., if upper limb function was not affected) is a key strategy used in strengthening mutual respect and intimacy. To reduce dependency-inducing behaviours and encourage an interdependent caregiving relationship, family caregivers must learn when to step back from caregiving tasks, a skill crucial in ensuring sustainable caregiving.

The participants in our study reported that constant care provision brought about psychological distress among caregivers, leading to caregivers not being able to intimately connect with their injured spouse. Particularly, the obligation to fulfill such caregiving duties and responsibilities on a daily basis was associated with the role change from spouse/partner to caregiver, and directly affected the loss of sex and intimacy in couples. A similar theme of “post-injury shift in relationship dynamics” (i.e., re-defining the spousal role) was determined by Dickson and colleagues [5] in their study focusing on the impact of assuming the primary caregiver role following traumatic spinal cord injury. The authors in that study identified that performing bodily tasks for the individuals with SCI (e.g., cleaning the individual after an ‘accident’ or emptying the colostomy bags), had a negative impact on the sexual relationship of couples. Consequently, this loss of sexual relationship reduced the former spousal or lover role to one where they were occupied with practical tasks for their injured spouse, and undertaking a more “motherly/fatherly” role [5]. Speziale [42] further reported that even slight adjustments in sexual intimacy can reduce the chance of maintaining ‘closeness’ and can result in strain within the spousal relationship. The similarities between our findings and other studies prompt the question of: if certain types of care activities (e.g., performing bodily tasks) reduce intimacy within relationships, should family caregivers assume responsibility for such tasks? Expanding the role of formal caregivers (e.g., personal support attendants) in performing certain care activities, such as bowel care (which hired caregivers are often unwilling or unable to perform [3]) may be a possible avenue to ensure sustainable intimate relationships.

Despite this, several of our participants did report that they were able to positively re-appraise the situation by utilizing innovative coping strategies within their caregiving relationships. Although developing a routine to manage caregiving tasks has been noted as an important skill to ensure competent caregiving, it also often leaves little room for creativity or spontaneity in romantic relationships. Indeed, Dickson and colleagues [5] reported that a lack of spontaneity can be detrimental to the family caregiver’s self-esteem (feelings of entrapment and invisibility) - perhaps a likely cause of difficulty in bonding with the care recipient. Role play, as a strategy to add creativity into routine caregiving tasks, was mentioned in the current study to cope with the loss of sexual relationship and shift in dynamics of the relationship. The participants in this study mentioned that this ‘trade-off’ of role interaction (using intimacy in caregiving) diminishes the perception of the ‘caregiver role’ and enhances the ‘spousal or lover role’. Role play or intimacy in caregiving is not a well-researched topic; nonetheless, this is an important aspect in caregiving that must be further explored to support couples adjusting to their romantic relationships post-injury.

A study by Kreuter and colleagues [13], that explored partner relationships, functioning, mood and global quality of life of individuals with SCI, identified several reasons for divorce. The reasons included difficulties adapting to new physical functions, challenges in maintaining the relationship, and/or unwillingness to live with the injured individual [13]. Our results further suggest that a dyad’s anticipation of the injured individual’s quick recovery during the early stage and hoping that the situation is temporary had led to maladaptive behaviours within their relationship. Wiles and colleagues [43], in their qualitative study on patients’ and carers’ expectations of recovery following stroke, identified that expectations of complete recovery may be a coping mechanism for patients and caregivers, which demonstrates a psychological need for optimism and hope. Although this may be true, our findings indicate that unrealistic recovery expectations by both individuals with SCI and their family caregivers resulted in the dyads not being able to cope with the new life post-injury, and losing sense of control over their future after the realization of the permanent nature of the injury. This led to resentment and withdrawal in dyads, and consequently disintegration of relationships. Furthermore, unlike stroke, the functionality of the injured individual does not improve over time [7], and the possible permanency of the injury [44] requires a dual effort by both individuals with SCI and their family caregivers in accepting or finding approaches to adapting to the new life post-injury.

Angel and Buss [24] suggested retaining some elements from the previous life as a possible strategy to adapting to the injury for individuals with SCI and their caregiving partners. Our findings also suggest a few participants attempted to continue prior activities (e.g., adapted travelling) to retain normality post-injury. Conversely, our findings also suggest creating a new normal by gradually establishing a new routine as a dyad as another approach to adapting to the new life post-injury. Indeed, this represents a synergy between creating a new normal and integrating some prior elements post-injury. The theme ‘creating a new normal’ is consistent with Strauss and colleagues’ [45] description of normalizing as a fundamental strategy among individuals with chronic diseases and illnesses. Feeling normal and attempts to normalize are vital concepts to individuals with SCI [46], and families continuously change their perceptions of ‘normal’ contingent on the injury and family situation [47]. Indeed, the participants in the current study strived to create a new normal by identifying alternative ways for the injured individuals to be active in day-to-day life based on their level of function which was characterized as being beneficial to the dyad and family as a unit (e.g., shifting from being the breadwinner to a domestic role). Chan’s [41] findings corroborated our study in that paraplegic men changed their role from being the primary earner in the family to taking on more household chores and providing care to children. The similarities between our findings and other studies indicate that creating a new normal requires care recipients and caregivers to simultaneously shift former social roles and norms, an aspect that has rarely been explored within caregiving literature in SCI.

### Limitations

Despite the strengths of the current study, a few possible limitations apply. With regard to the recruitment of participants, it is possible that a selection bias may have occurred. It is likely that those participants who agreed to participate may have been better adjusted to injury than those individuals who declined participation. This is possibly a reason for an over-representation in the over 10 years post-discharge group as they have had more time to adapt to life after the injury. Discharge from inpatient rehabilitation is a crucial time for the caregivers of individuals with SCI due to apprehension of taking on a new role which may result in adjustment challenges within relationships. More research exploring the experiences and challenges of individuals with SCI and their family caregivers during the initial stage of transitioning back home is necessary to ensure sustainability within relationships. Also, the majority of family caregivers in the current study were females. Future research should be directed towards exploring the experiences of male caregivers and its impact on relationship dynamics and marital adjustments. Such knowledge could be used to increase the relevance of care models and programs for both male and female caregivers.

### Implications for practice and service provision

Evidence-based interventions are needed to help individuals with SCI and their family caregivers adjust to the many personal and interpersonal challenges experienced post-injury. A recent study by Molazem and colleagues [48] reported that the quality of life (including physical function, social function, role emotional and mental health) had improved in family caregivers who received psycho-educational interventions (e.g., educational sessions on coping strategies, crisis confrontation strategies, appropriate care provision to the care recipient, etc.) in comparison to the usual care control group (i.e., did not receive educational sessions). Although available interventions show optimized quality of life for family caregivers, there is a lack of intervention studies particularly on optimizing caregiving relationships post-SCI. Findings from this study suggest the need for relationship-based education; particularly, communication skills training, to help dyads living with SCI to manage challenges, negotiate changes, and facilitate positive interactions within their relationships. Communication styles that previously worked in relationships may not be successful post-injury. Furthermore, counseling during rehabilitation could educate and prepare individuals with SCI and family caregivers for the challenges that may arise in daily life post-discharge into community. Chan [41] noted the importance of considering the dyad as a single unit to promote increased understanding and preparedness post-injury. The possible value of formal support from healthcare professionals to help care recipients and caregivers to negotiate improvements in communication within their relationship could potentially reduce long-term issues, including irreparable damages in the nature of their relationship. In combination with professional support, connecting family caregivers with peer support groups (i.e., matched peer mentor and mentee) could also help prepare them for their caregiving role, as well as reduce feelings of loneliness and social isolation and psychological distress [29, 49] Furthermore, the timing of emotional or psychological support is vital to developing a system that is responsive to caregivers’ ‘readiness’ to receive such specific support [50]. Although readiness to receive emotional or psychological support is dependent on the individual’s adjustment process [51], the findings from the current study suggest the possible value of offering counseling and peer support prior to discharge from inpatient rehabilitation or early in the transition to the community. This would ensure relevant support for individuals with SCI and families to help them maintain the stability of relationships post-discharge (i.e., during the first year post-discharge), a time period that can be associated with family dissolution or even divorce [29].

## Conclusion

Overall, this study demonstrated that individuals with SCI and their caregiving partners experience a range of emotional, psychological, and relationship challenges post-injury. However, collaborating as a dyadic unit, care recipients and caregivers could negotiate these challenges and changes by devising coping strategies to sustain their relationships. These findings should further alert healthcare professionals (and/or peer support groups) as to the need for possible education and training (e.g., communication skills training, coping strategies) as well as counseling to prepare dyads to negotiate changes within their relationships post-discharge. This study is an important first step in advancing exploratory research about the factors that challenge the stability of relationships as well as various dyadic coping strategies used by individuals with SCI and their family caregivers in fostering healthy caregiving relationships.

## Data Availability

The datasets generated and/or analyzed during the current study are not publicly available due to the existing parameters of our REB application/approval but are available from the corresponding author on reasonable request and pending research ethics approval.

## Abbreviations

ADL: Activities of daily living
RHISCICS: Rick Hansen Institute SCI Community Survey
SCI: Spinal cord injury
SCIO: Spinal Cord Injury Ontario
TBI: Traumatic brain injury
TDF: Theoretical Domains Framework
TRI: Toronto Rehabilitation Institute
